# Coding-Complete Genome Sequences and Mutation Profiles of Nine SARS-CoV-2 Strains Detected from COVID-19 Patients in Bangladesh

**DOI:** 10.1128/MRA.00124-21

**Published:** 2021-03-11

**Authors:** Nihad Adnan, Mohib Ullah Khondoker, M. Shaminur Rahman, M. Firoz Ahmed, Shahana Sharmin, Nadim Sharif, Nafisa Azmuda, Salma Akter, Shamsun Nahar, Taslin Jahan Mou, Mahfuza Marzan, Syeda Moriam Liza, Nowshin Jahan, Tamanna Ali, Shahad Saif Khandker, Maha Jamiruddin, Mousumi Akter Chaity, Laura Grace Caller, M. Ahsanul Haq, Mohd Raeed Jamiruddin

**Affiliations:** a Department of Microbiology, Jahangirnagar University, Savar, Dhaka, Bangladesh; b Gonoshasthaya-RNA Molecular Diagnostic and Research Center, Dhanmondi, Dhaka, Bangladesh; c Gonoshasthaya Samaj Vittik Medical College, Savar, Dhaka, Bangladesh; d Center for Multidisciplinary Research, Gono Bishwabidyalay, Savar, Dhaka, Bangladesh; e Department of Microbiology, University of Dhaka, Dhaka, Bangladesh; f Department of Pharmacy, BRAC University, Dhaka, Bangladesh; g Department of Pathology, University of Cambridge, Cambridge, United Kingdom; Queens College

## Abstract

Here, we report the coding-complete genome sequences of nine clinical severe acute respiratory syndrome coronavirus 2 (SARS-CoV-2) variants and their mutations. The samples were collected from nine Bangladeshi coronavirus disease 2019 (COVID-19) patients. We have identified the E484K escape mutation and the S359T mutation within the spike protein coding region of the sequenced genomes.

## ANNOUNCEMENT

Severe acute respiratory syndrome coronavirus 2 (SARS-CoV-2), a member of the *Betacoronavirus* genus in the *Coronaviridae* family, has been responsible for more than 2 million deaths globally (1). In this study, we performed coding-complete genome sequencing of nine clinical SARS-CoV-2 isolates to observe genomic and inferred proteomic mutational variations (Table 1). The samples were collected at Gonoshasthaya-RNA Molecular Diagnostic and Research Center (Dhaka, Bangladesh) within the period from October to December 2020, with ethical clearance from the National Research Ethics Committee (approval number BMRC/NREC/2019-2022/697). The samples were identified as positive by the novel coronavirus (2019-nCoV) nucleic acid diagnostic kit (Sansure, Inc., China) and had cycle threshold (*C_T_*) values ranging from 27 to 30, which, according to the kit’s information, implies high viral load.

**TABLE 1:** Genomic features of nine SARS-CoV-2 clinical samples

Sample no.	BioSample no.	SRA accession no. (raw reads)	GenBank accession no.	Total no. of reads	No. of mapped reads	Avg coverage (×)	Assembly length (bp)	GC content (%)	PANGO lineage	Emerging clade
GRBL_S1	SAMN17359583	SRR13449688	MW532093	326,252	324,957	39.98	29,847	38.01	B.1.1.103	20B
GRBL_S2	SAMN17359584	SRR13449687	MW532094	7,951,736	7,940,015	974.5	29,858	38.01	B.1.1.103	20B
GRBL_S3	SAMN17359585	SRR13449686	MW532095	208,394	203,220	25.54	29,842	38.03	B.1.1.103	20B
GRBL_S4	SAMN17359586	SRR13449685	MW532096	1,526,918	1,473,009	187.13	29,856	38.01	B.1.1.103	20B
GRBL_S6	SAMN17359587	SRR13449684	MW532097	353,644	345,476	43.34	29,823	38.37	B.1.1.316	20B
GRBL_S9	SAMN17359588	SRR13449683	MW532098	6,758,972	6,749,017	828.32	29,863	37.99	B.1.1.316	20B
GRBL_S10	SAMN17359589	SRR13449682	MW532099	126,716	125,886	15.53	29,850	38.00	B.1.1.25	20B
GRBL_S11	SAMN17359590	SRR13449681	MW532100	11,244,412	11,228,723	1,378.02	29,868	37.99	B.1.1.25	20F
GRBL_S14	SAMN17359592	SRR13449679	MW532101	151,570	150,440	18.58	29,849	38.00	B.1.1.316	20B

The genomic viral RNA was purified from both nasopharyngeal and oropharyngeal swabs from individuals (both male and female patients; age range, 17 to 68 years) suspected of being infected with SARS-CoV-2, using the ReliaPrep viral TNA miniprep system (Promega) according to the manufacturer’s instructions. This was followed by preparation of libraries using Illumina RNA preparation with enrichment in combination with the Illumina respiratory virus oligonucleotide panel v2 according to the manufacturer’s instructions (Illumina, Inc., San Diego, CA). The sequencing was carried out in an Illumina MiniSeq instrument implementing a paired-end protocol (read length, 74 bp). The Fastq sequences were trimmed, quality controlled, and mapped and a consensus sequence was generated using DRAGEN v3.5.1.15 (Illumina) (2). The genome coverages and mutations were initially checked with SAMtools and Snippy in comparison with the Wuhan reference genome (GenBank accession number MN996528) (3–6). The novel mutations were confirmed with the EpiCov tool, integrated in the Global Initiative on Sharing All Influenza Data (GISAID) database (7). All mutations and deletions were confirmed by area-wise coverage using Snippy (5).

**FIG 1:**
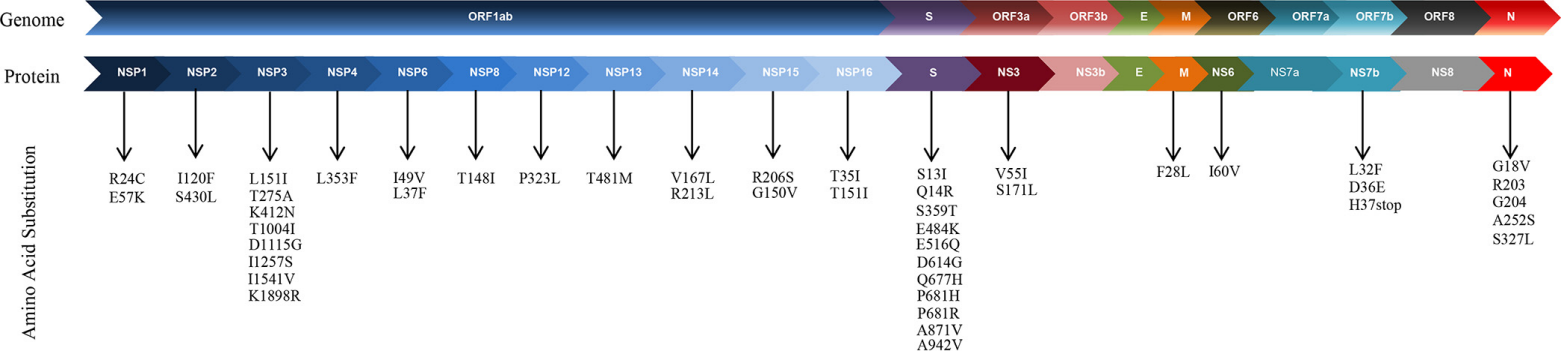
Complete list of amino acid substitutions in the genomes of nine different clinical SARS-CoV-2 isolates, with reference to isolate WIV04 (GenBank accession number MN996528).

Eight strains (GRBL_S1, GRBL_S2, GRBL_S3, GRBL_S4, GRBL_S6, GRBL_S9, GRBL_S10, and GRBL_S14) belong to the emerging clade 20B, whereas one strain (GRBL_S11) is affiliated with the 20F clade (Table 1). In this announcement, among the multitude of mutations, we are reporting two mutations, one at position 1450 from G (0 evidence) to A (267 evidence) in GRBL_S1, which results in the amino acid substitution of E484K, and one at position 1076 from G (0 evidence) to C (4,559 evidence) in GRBL_S9, which results in S359T in the spike protein region (“evidence” relates to area-wise coverage obtained by Snippy tools for short-read sequences generated by Illumina MiniSeq). The former mutation was reported in the South African variant as an escape mutation (8, 9). The E484K substitution was observed in a cluster containing D614G, P681H, and S13I changes (GRBL_S1), while the S359T amino acid substitution was observed in a cluster containing D614G and A942V changes (GRBL_S9). Additionally, we found cluster substitutions D614G, Q677H, and A871V in the spike protein of GRBL_S10. Within the open reading frame 1ab (ORF1ab) region, we observed amino acid substitutions of I1257S in GRBL_S1 and L151I and T275A in GRBL_S14. The complete list of amino acid substitutions is provided in Fig. 1.

It is also noteworthy that in two samples, GRBL_S2 (2,446 evidence) and GRBL_S3 (68 evidence), the deletion of GATCAT and its subsequent replacement by G at nucleotide position 108 of ORF7b resulted in the introduction of a stop codon, ultimately resulting in a frameshift mutation and D36E substitution, potentially without any loss of function (10). The patients harboring these variants were detected as family members residing within the same household. Substitutions pertaining to the nucleocapsid protein coding region were S327L, G18V, and A252S in samples GRBL_S1, GRBL_S11, and GRBL_S14, respectively. Further investigations are required to identify the effects of the substitutions on the outcomes of coronavirus disease 2019 (COVID-19).

### Data availability.

The sequences of nine SARS-CoV-2 genomes were submitted to the GISAID database under the identifiers EPI_ISL_774976, EPI_ISL_775019, EPI_ISL_775020, EPI_ISL_890189, EPI_ISL_775215, EPI_ISL_775218, EPI_ISL_890192, EPI_ISL_890193, and EPI_ISL_890194 and to the NCBI GenBank under the accession numbers MW532093, MW532094, MW532095, MW532096, MW532097, MW532098, MW532099, MW532100, and MW532101 (Table 1). The raw reads were submitted to the NCBI SRA under BioProject accession number PRJNA692653 and SRA accession number SRP302071.

## References

[1] World Health Organization. 2020. WHO coronavirus disease (COVID-19) dashboard. https://covid19.who.int. Accessed 31 January 2021.

[2] Goyal A, Kwon H, Lee K, Garg R, Yun S, Hee Kim Y, Lee S, Seob Lee M. 2017. Ultra-fast next generation human genome sequencing data processing using DRAGEN^TM^ Bio-IT processor for precision medicine. Open J Genet 7:9–19. doi:10.4236/ojgen.2017.71002.

[3] Li H, Handsaker B, Wysoker A, Fennell T, Ruan J, Homer N, Marth G, Abecasis G, Durbin R. 2009. The Sequence Alignment/Map format and SAMtools. Bioinformatics 25:2078–2079. doi:10.1093/bioinformatics/btp352. 19505943 PMC2723002

[4] Etherington GJ, Ramirez-Gonzalez RH, MacLean D. 2015. bio-samtools 2: a package for analysis and visualization of sequence and alignment data with SAMtools in Ruby. Bioinformatics 31:2565–2567. doi:10.1093/bioinformatics/btv178. 25819670

[5] Seeman T. 2015. Snippy: rapid haploid variant calling and core SNP phylogeny. https://github.com/tseemann/snippy.

[6] Koyama T, Platt D, Parida L. 2020. Variant analysis of SARS-CoV-2 genomes. Bull World Health Organ 98:495–504. doi:10.2471/BLT.20.253591. 32742035 PMC7375210

[7] Elbe S, Buckland-Merrett G. 2017. Data, disease and diplomacy: GISAID's innovative contribution to global health. Glob Chall 1:33–46. doi:10.1002/gch2.1018. 31565258 PMC6607375

[8] Tegally H, Wilkinson E, Giovanetti M, Iranzadeh A, Fonseca V, Giandhari J, Doolabh D, Pillay S, San EJ, Msomi N, Mlisana K, von Gottberg A, Walaza S, Allam M, Ismail A, Mohale T, Glass AJ, Engelbrecht S, Van Zyl G, Preiser W, Petruccione F, Sigal A, Hardie D, Marais G, Hsiao M, Korsman S, Davies M-A, Tyers L, Mudau I, York D, Maslo C, Goedhals D, Abrahams S, Laguda-Akingba O, Alisoltani-Dehkordi A, Godzik A, Wibmer CK, Sewell BT, Lourenço J, Alcantara LCJ, Pond SLK, Weaver S, Martin D, Lessells RJ, Bhiman JN, Williamson C, de Oliveira T. 2020. Emergence and rapid spread of a new severe acute respiratory syndrome-related coronavirus 2 (SARS-CoV-2) lineage with multiple spike mutations in South Africa. medRxiv 2020.12.21.20248640. doi:10.1101/2020.12.21.20248640.

[9] Collier DA, De Marco A, Ferreira IATM, Meng B, Datir R, Walls AC, Kemp SA, Bassi J, Pinto D, Fregni CS, Bianchi S, Tortorici MA, Bowen J, Culap K, Jaconi S, Cameroni E, Snell G, Pizzuto MS, Pellanda AF, Garzoni C, Riva A, Elmer A, Kingston N, Graves B, McCoy LE, Smith KG, Bradley JR, Thaventhiran JJ, Ceron-Gutierrez L, Barcenas-Morales G, Virgin HW, Lanzavecchia A, Piccoli L, Doffinger R, Wills M, Veesler D, Corti D, Gupta RK. 2021. SARS-CoV-2 B.1.1.7 escape from mRNA vaccine-elicited neutralizing antibodies. medRxiv 2021.01.19.21249840. doi:10.1101/2021.01.19.21249840.

[10] Su YCF, Anderson DE, Young BE, Linster M, Zhu F, Jayakumar J, Zhuang Y, Kalimuddin S, Low JGH, Tan CW, Chia WN, Mak TM, Octavia S, Chavatte JM, Lee RTC, Pada S, Tan SY, Sun L, Yan GZ, Maurer-Stroh S, Mendenhall IH, Leo YS, Lye DC, Wang LF, Smith GJD. 2020. Discovery and genomic characterization of a 382-nucleotide deletion in ORF7b and ORF8 during the early evolution of SARS-CoV-2. mBio 11:e01610-20. doi:10.1128/mBio.01610-20. 32694143 PMC7374062

